# Using Cy5-dUTP labelling of RPA-amplicons with downstream microarray analysis for the detection of antibiotic resistance genes

**DOI:** 10.1038/s41598-021-99774-z

**Published:** 2021-10-11

**Authors:** Christian Warmt, Carolin Kornelia Fenzel, Jörg Henkel, Frank Fabian Bier

**Affiliations:** 1 Fraunhofer Institute for Cell Therapy and Immunology - Bioanalytics and Bioprocesses (IZI-BB), 14476 Potsdam, Germany; 2 Institute for Biochemistry and Biology, University of Potsdam, 14476 Potsdam, Germany; 3 Institute for Molecular Diagnostics and Bioanalysis, IMDB, 16761 Hennigsdorf, Germany

**Keywords:** Biological techniques, Molecular biology

## Abstract

In this report we describe Cy5-dUTP labelling of recombinase-polymerase-amplification (RPA) products directly during the amplification process for the first time. Nucleic acid amplification techniques, especially polymerase-chain-reaction as well as various isothermal amplification methods such as RPA, becomes a promising tool in the detection of pathogens and target specific genes. Actually, RPA even provides more advantages. This isothermal method got popular in point of care diagnostics because of its speed and sensitivity but requires pre-labelled primer or probes for a following detection of the amplicons. To overcome this disadvantages, we performed an labelling of RPA-amplicons with Cy5-dUTP without the need of pre-labelled primers. The amplification results of various multiple antibiotic resistance genes indicating great potential as a flexible and promising tool with high specific and sensitive detection capabilities of the target genes. After the determination of an appropriate rate of 1% Cy5-dUTP and 99% unlabelled dTTP we were able to detect the *bla*_CTX-M15_ gene in less than 1.6E−03 ng genomic DNA corresponding to approximately 200 cfu of *Escherichia coli* cells in only 40 min amplification time.

## Introduction

Whereas polymerase chain reaction (PCR) is a common technique for the amplification of DNA fragments and a widely used nucleic acid amplification technology (NAAT) for the detection and differentiation of pathogens^1^ as well as for the specific detection of resistance genes^2^, isothermal amplification methods such as recombinase polymerase amplification (RPA) getting more and more attention and popularity for NAAT^3^.

There are some crucial disadvantages for the usage of the PCR in the field of lab-on-chip or point-of-care (PoC) testing including the necessity of a fast and accurate temperature cycling process that requires high performance power supply’s and a high demand of energy. Furthermore, the amplification time can take up to two or three hours until sufficient amounts of amplicons are produced for the detection^4^ and additionally the need of temperatures with up to 98 °C during cycling steps that ensures DNA denaturation and DNA strand unwinding.

To overcome previous named disadvantages, isothermal amplification methods are promising tools as alternatives to the commonly used PCR technique^5^. These techniques use different strategies to avoid temperature cycling and the denaturation problem^6,7^.

Isothermal amplification methods are used more often and representing more simplified handling in contrast to the PCR using additional enzymes and binding proteins^5^.

RPA for example combines the usage of recombinase and single strand binding proteins (SSB) with a strand displacement polymerase^6^.

Amplification times of less than 20–30 min are possible^8^ and there is no need for expensive equipment. Crannel et al.^9^ has shown that RPA already works with 37 °C body temperature using the armpit.

The combination of RPA with various subsequent detection methods is very easy and enables a broad range of applications^3^. The most commonly used one is the combination with some kind of lateral flow assays^10,11^. More than 40% of the published results in 2020 used lateral flow based detection of RPA amplicons. Nevertheless CRISPR-Cas based systems^12^ are used in the same way like colorimetric assays^13^, gel electrophoresis^10^, electrochemical detection systems^14,15^ and microarray based techniques^16^.

Using techniques like lateral flow or quantitative assays as well as microarrays, labelling of the amplicons are essential for the detection. This is commonly achieved by pre-labelled oligonucleotides^11,16^.

Unfortunately, using pre-labelled primer and probes is often less flexible and expensive in assays with huge sets of up to tens and hundreds of oligonucleotides. Every new primer set has to be labelled before experiments, for example with fluorescent molecules.

Furthermore, there is no potential to adjust the signal intensity by adjusting the amount of fluorophores. Using pre-labelled primer you can attach only one fluorophore to one DNA single strand. Additionally, changing the labelling and detection method requires new sets of primer with the new labelling system.

These gave us the motivation to investigate a direct and flexible labelling of RPA amplicons with labelled nucleotides instead of primers. This gives us more flexibility without pre-labelled primer sets.

**Figure 1:**
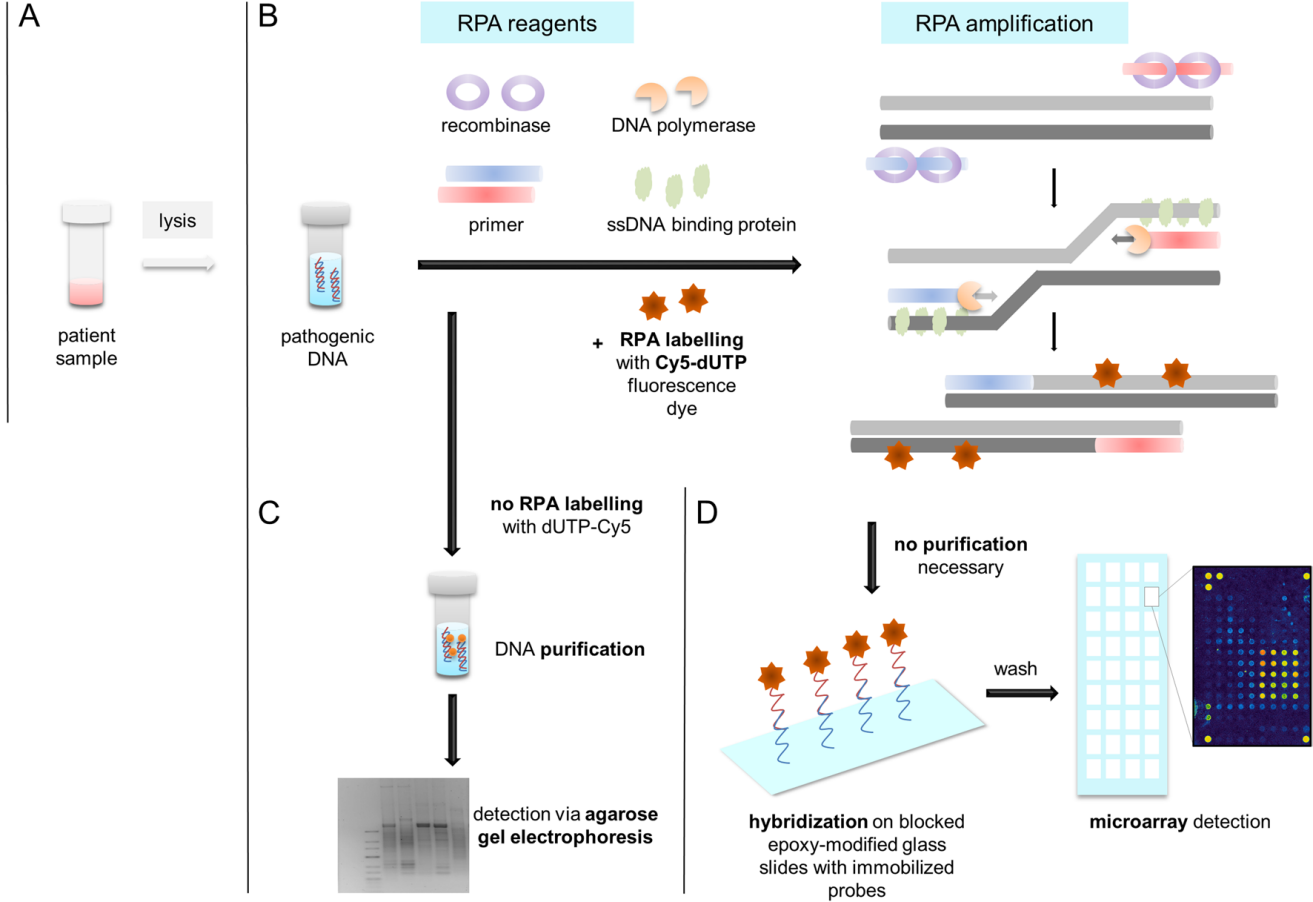
Overview of the experimental workflow. Pre-preparation and extraction of the DNA (**A**) are not included in this study. (**B**) For the amplification of the pathogenic DNA, recombinase polymerase amplification (RPA) was performed using Cy5-dUTP for labelling of the products. (**C**) After the RPA the amplicons has to be purified for detection and validation via agarose gel electrophoresis. (**D**) For the hybridization and subsequent microarray detecting of the pre-labelled amplicons, no purification is necessary. This step was performed using immobilized probes on epoxy-modified glass slides.

Aim of this study is the development of an alternative labelling technique for a fast and sensitive amplification and detection of multidrug resistance genes, that are still a health concern of key importance and represents a serious problem^2,17,18^, based on recombinase polymerase amplification with subsequent hybridization and microarray detection (Fig. 1).

For this, we used 5-(3-Aminoallyl)-2′-deoxyuridine-5′-triphosphate (Cy5-dUTP) for labelling of RPA amplicons during the amplification process. A few studies are existing using this technique for labelling PCR products with fluorophores^19–21^ and other molecules like biotin^21,22^, but despite some advantages this is not a commonly used method.

This is a proof of concept study for direct labelling of RPA amplicons with labelled nucleotides using fluorophores for the subsequent detection via microarray analysis.

As far as we know, this is the very first time a direct labelling method by using modified nucleotides, shown in this study with Cy5-dUTP, is mentioned in the literature for an incorporation of fluorophores during the isothermal method RPA.

## Results

### Finding the appropriate Cy5-dUTP amount

**Figure 2:**
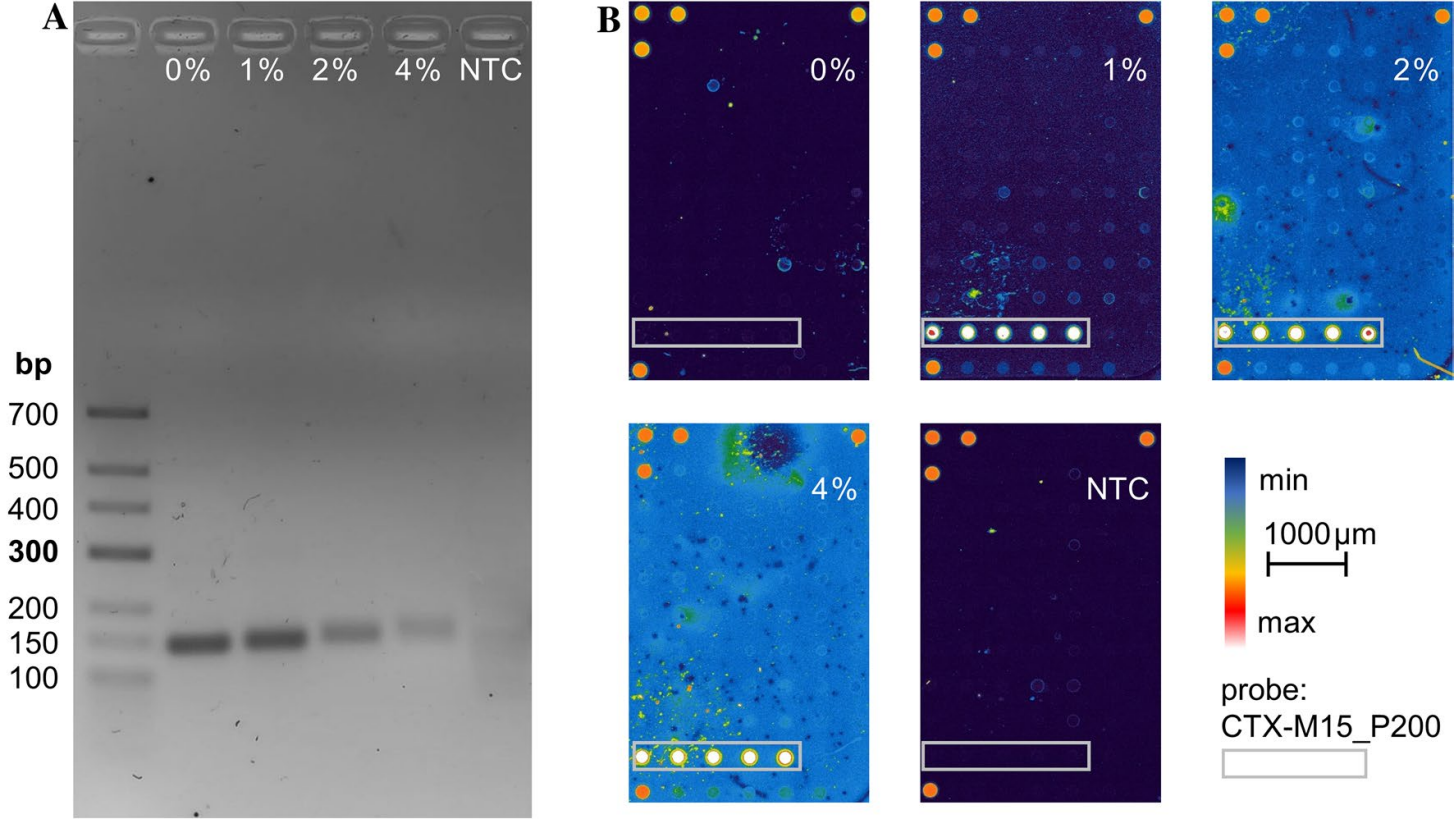
Determination of the suitable Cy5-dUTP amount. 40 min RPA (38 °C) was performed using the resistance gene *bla*_CTX-M15_ in *E. coli* genomic DNA samples as target. (**A**) Gel electrophoresis with purified amplicons after RPA with different Cy5-dUTP amounts compared to the unlabelled dTTP. (NTC: no template control). (**B**) Hybridization and microarray detection of the RPA products shown in (**A**). (grey box indicating the position of the *bla*_CTX-M15_ specific probe; pictures are false colour displayed, NTC: no template control; scan power 60% PMT gain 530, five dots in the three corners indicating spotting controls).

*Escherichia coli* genomic DNA was used with a known encoded *bla*_CTX-M15_ resistance gene for RPA labelling with various amounts of Cy5-dUTP to determine a suitable ratio for the RPA. Observing purified amplicons by gel electrophoresis (Fig. 2A) we were able to detect products using a ratio of 1–4% labelled nucleotides and 99%-96% unlabelled ones.

Comparison of various amounts of fluorescent nucleotides shows no inhibition of RPA using only 1% Cy5-dUTP whereas experiments with 4% showing only very small amounts of target DNA. On the other hand, we could assess that RPA amplicons with the same target size produces a small electrophoretic gel shift with an increased amount of Cy5-dUTP.

We also performed a hybridization of the unpurified RPA products using *bla*_CTX-M15_ specific probes immobilized on a 3D-epoxy glass surface (Fig. 2B). Besides *bla*_CTX-M15_, several probes for various resistance genes were immobilized on the microarray and used in subsequent experiments. Only the target specific ones are visible after the hybridization with the labelled amplicons. We observed that the 1% Cy5-dUTP labelling generates sufficient but still the lowest signal intensities (indicated by the colour spectrum of the false colour display) whereas 2% and 4% Cy5-dUTP producing higher signals despite the lower amounts of RPA products seen by gel electrophoresis. On the other hand, there is a vast increase of the background signal using 4% labelling compared to 1% represented by the colour change of the arrays from deep blue (normal RPA without labelling) to light blue by using 4% Cy5-dUTP.

The orange coloured dots in the three corners (upper left, lower left and upper right) representing the pre-labelled spotting control DNA and indicating the orientation of the array.

Based on the results shown, a ratio of 1% Cy5-dUTP / 99% dTTP for labelling was used in further experiments.

### Detecting various resistance genes

In the second part of the experiments, we applied the method developed with *bla*_CTX-M15_, using 1% Cy5-dUTP, to further resistance genes. We tried to detect not only *bla*_CTX-M15_ but *bla*_KPC_ and *bla*_NDM_ as well as *bla*_VIM_ via hybridization on a microarray subsequent to the recombinase polymerase amplification labelling.

For this, *E. coli* genomic DNA of two different strands for the *KPC* and *CTX-M15* assay as well as for *NDM* assay and genomic DNA from *P. aeruginosa* for *VIM* was used.

Our microarray did contain one specific probe for *bla*_CTX-M15_ (CTX-M15_P200), *bla*_NDM_ (NDM_P_new) and *bla*_VIM_ (VIM_P2_as). For the detection of *bla*_KPC_ a set of 4 probes only differing in a single mutation to determine whether it is possible to detect not only *bla*_KPC_ but the correct *KPC* variant was used. Additionally, a set of probes as negative hybridization controls which should only indicate the presence of other resistance genes were used.

**Figure 3:**
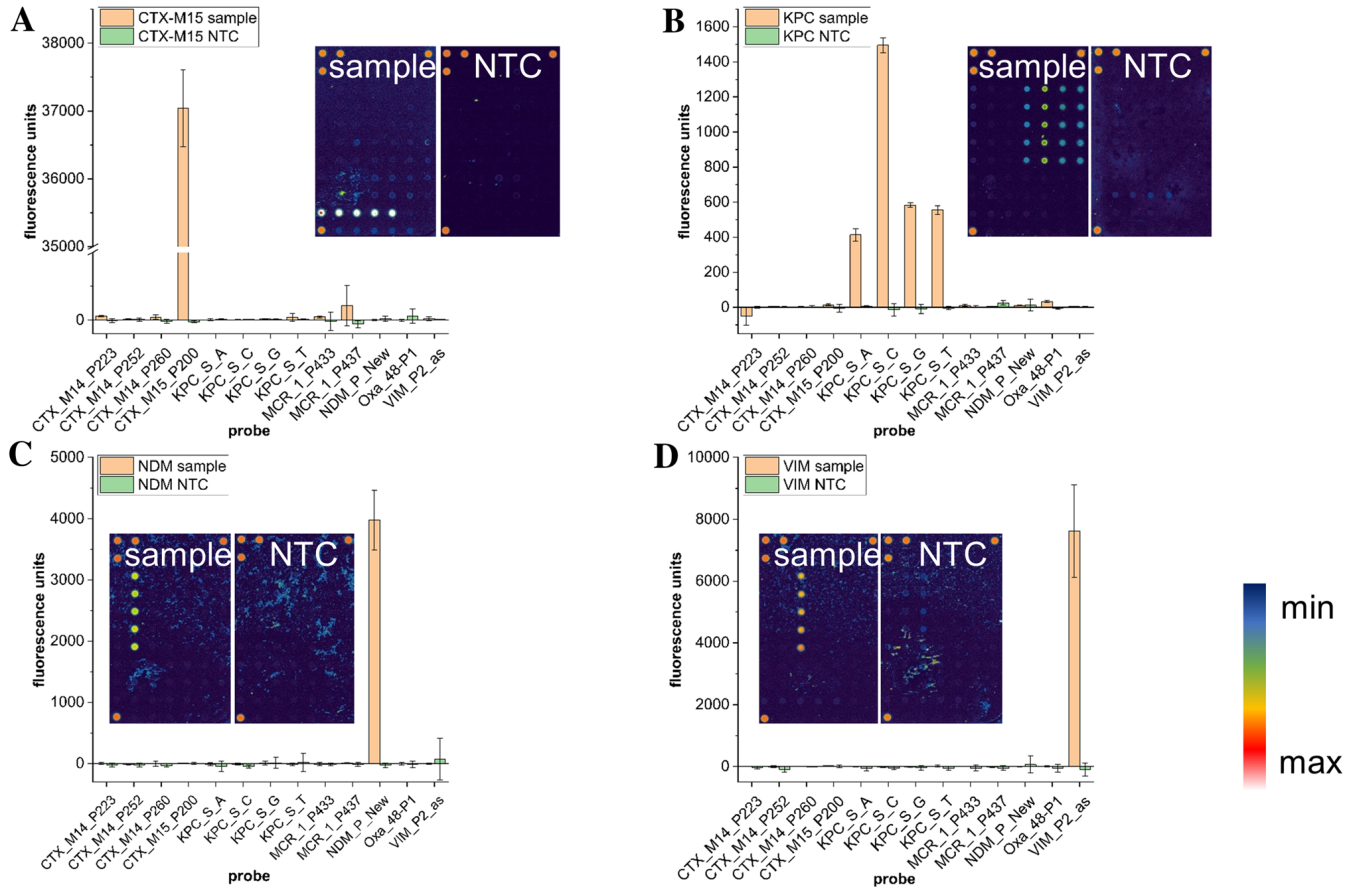
Detection of the resistance genes *bla*_CTX-M15_, *bla*_KPC_, *bla*_NDM_ and *bla*_VIM_. 40 min RPA (38 °C) was performed with genomic DNA using the following resistance genes and organisms as target: (**A**) *bla*_CTX-M15_, (**B**) *bla*_KPC_ and (**C**) *bla*_NDM_ (*E. coli*); (**D**) *bla*_VIM_ (*P. aeruginosa*). (**A**)–(**D**) demonstrating microarrays as well as graphical visualization of samples containing the genomic DNA (sample) and a no template control (NTC). Specific probes are CTX-M15_P200 (*bla*_CTX-M15_), KPC_S_A/C/G/T (*bla*_KPC_), NDM_P_new (*bla*_NDM_) and VIM_P2_as (*bla*_VIM_) (pictures are false colour displayed; scan power 60% PMT gain 530; error bar = standard deviation; n = 5; five dots in the three corners indicating spotting controls).

We exclusively noticed specific fluorescence signals for our targets (Fig. 3) and no false positives in the unspecific control probes or in the no template controls (NTC).

The false colour displayed array pictures clear arrays with very low background (deep blue array area). Probes do give fluorescence signals at different positions on the array indicating different fluorescent signal intensities. The intensities of the various resistance genes were found in the following order: *bla*_KPC_ < *bla*_NDM_ < *bla*_VIM_ < *bla*_CTX-M15_.

By observing and comparing the signal intensities determined under the same experimental conditions, we could see that the mean intensities range from 1.500 fluorescence units (fu) for the high signal *KPC* probe over 4.000 fu and 8.000 fu for the detection of *bla*_NDM_ and *bla*_VIM_ respectively up to 37.000 fu for *bla*_CTX-M15_. Having a look on the number of base pairs produced during RPA (Table 1) for each fragment it is noticeable that the observed signal intensities increases with the decrease of the fragment length. The *CTX-M15* fragment is the shortest one with 141 nucleotides (nt) producing the highest signal intensities followed by the *VIM* fragment with 191 nt and *NDM* with 220 nt. The RPA product for the *bla*_KPC_ detection is the longest one (809 nt) and produces the lowest signals with less than 2.000 fu for the high signal probe (KPC_S_C) and approximately 500 fu for the other ones (KPC_S_A/G/T).

**Table 1:** List of organisms and primers.

organism	Isolate no	Resistance gene	Primer	Sequence	Fragment (bp)
*E. coli*	2/10	*NDM-1*	NDM-R	CAAGCTGGTTCGACAACGCATTGGCAT	220
			NDM-F	CAACGGTTTGATCGTCAGGGATGGCGG	
*P. aeruginosa*	359/11	*VIM-2*	VIM-F	TGGTCTCATTGTCCGTGATGGTGATGAGTTGCT	191
			VIM-R	TACGTTGCCACCCCAGCCGCCCGAAGGACATC	
*E. coli*	17/11	*KPC-2*	KPC-F	CATTCGCTAAACTCGAACAGGACTTTG	809
			KPC-R	CCAATAGATGATTTTCAGAGCCTTACTG	
*E. coli*	735/14–1	*CTX-M15*	CTX-M15-F	TCACGCTGTTGTTAGGAAGTGTGCCGCTGTATGC	141
			CTX-M15-R	CGATAAAGTATTTGCGAATTATCTGCTGTGT	

Illustration of the organisms used in this study including the target resistance gene and the RPA primers. The isolate number refers to the nomenclature of the german Robert Koch Institute. All primers were synthesized and provided by metabion international AG.

Within this assay, we not only tried to detect *bla*_KPC_ itself but to determine the *KPC* variant by the high signal probe. The probe KPC_S_C was designed to detect the variant *KPC-2*, having a single nucleotide polymorphism (SNP) at position 308 of the *bla*_KPC_ gene ^23^. Both the false colour display (greenish colour) and the corresponding graphical evaluation indicates a higher fluorescence intensity of the KPC_S_C probe compared to the other three ones in this set.

### Determination of the assay sensitivities

We used a dilution series of the *E. coli* genomic DNA containing the *bla*_CTX-M15_ and *bla*_NDM_ resistance gene in a range from 1 to 6.4E−05 ng to evaluate the sensitivity of our novel RPA labelling method. The examined DNA concentration range corresponds to a number of 2.0E+05 cells up to approximately 10 cells, respectively. As before, the RPA was performed by using a ratio of 1% Cy5-dUTP/ 99% unlabelled dTTP.

**Figure 4:**
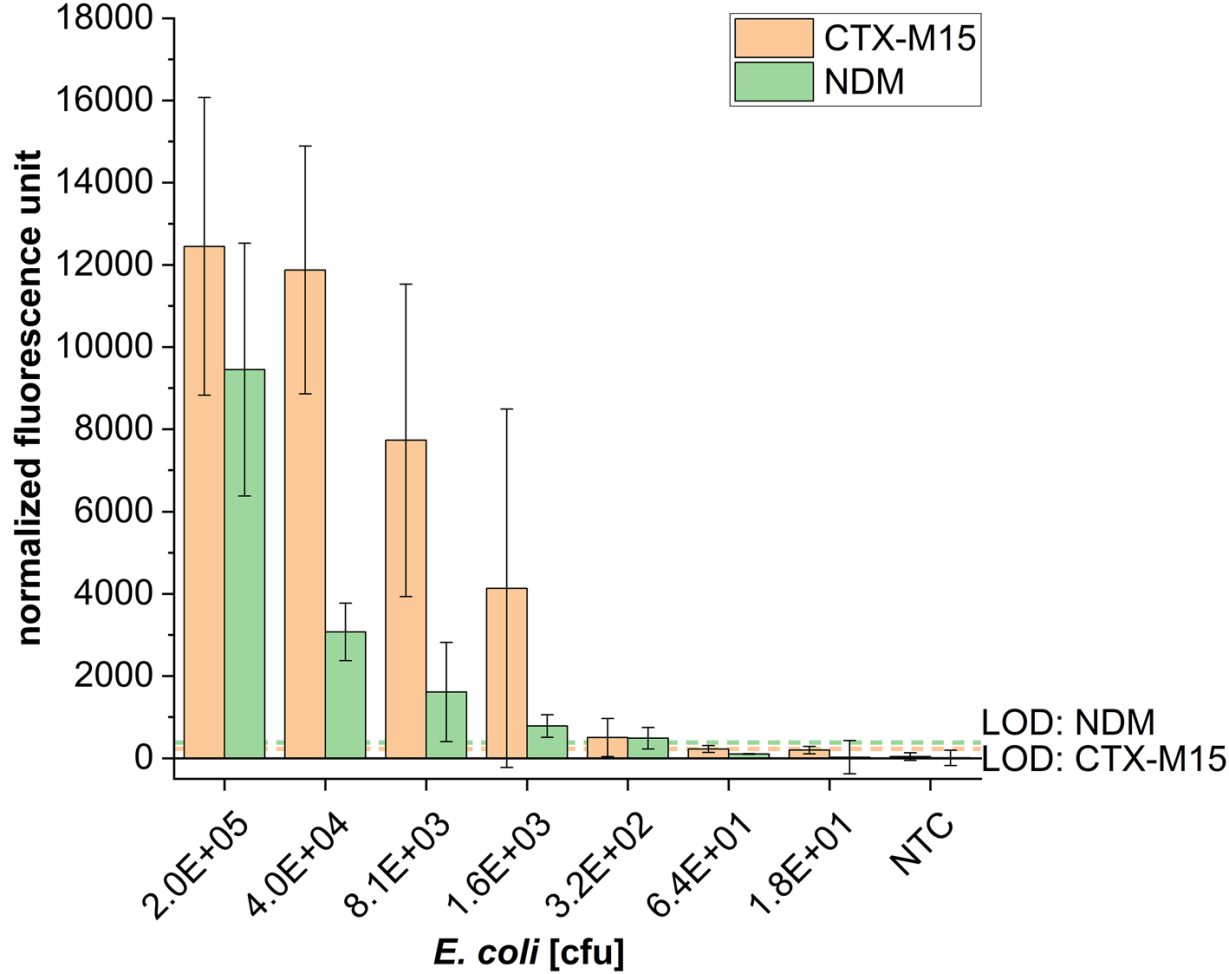
LOD determination for the genes *bla*_CTX-M15_, and *bla*_NDM_. 40 min RPA (38 °C) was performed with genomic DNA using *bla*_CTX-M15_ and *bla*_NDM_ in two different *E. coli* strands. Dilution series of *E. coli* genomic DNA from 1 ng up to 6.4E−05 ng genomic DNA corresponds to the indicated cell numbers (x-axis). Fluorescence intensity are normalized to the mean signal intensities of the hybridization control in each array. The limit of detection (LOD) was calculated using the sum of the fluorescence signal of the NTC and the two fold standard deviation. Specific probes are CTX-M15_P200 (*bla*_CTX-M15_) and NDM_P_new (*bla*_NDM_) (error bar = standard deviation; n = 6).

Additionally, a hybridization control with a known and consistent concentration during the microarray step was used. The fluorescence intensities of the hybridization controls are illustrated in Fig. 4.

For the graphical evaluation (Fig. 4) the fluorescence intensities of the gene target was normalized to the fluorescence intensity of the standardised hybridization control. For this, the intensities of the control and the targets within the array containing the highest DNA concentration was set to 100%. The target intensities of the remaining concentrations were multiplied by the quotient of the control intensities of this array and the control intensities of the reference assay (normalized fluorescence units, nfu).

With this we were able to detect more than 12.000 nfu and 9.000 nfu for 2.0E+05 cells containing *bla*_CTX-M15_ and *bla*_NDM_ resistance genes respectively. This fluorescence intensity decreases with different strength for *bla*_NDM_ and *bla*_CTX-M15_ by decreasing the number of cells used for the RPA. Using less than 1000 cells for the amplification process, the intensities decreases to approximately 500 nfu for 300 cells as well as 224 nfu and 102 nfu for *CTX-M15* and *NDM* respectively for 64 cells.

Taking the fluorescence of the no template controls plus the two fold standard deviation we calculated a limit of detection (LOD) of 228 cells for the *CTX-M15* assay and 380 cells for the *NDM* assay.

## Discussion

In this study we showed the incorporation of 5-(3-Aminoallyl)-2′-deoxyuridine-5′-triphosphate (Cy5-dUTP) by the polymerase *Bsu*^6^ directly during the RPA process.

For PCR-labelling amounts of 30–50% Cy5-dUTP/70-50% unlabelled dTTP is recommended^24^. However, we observed that in PCR 10% Cy5-dUTP is sufficient for effective labelling (data not shown).Therefor we tried to figure out the best Cy5-dUTP/dTTP ratio for usage with recombinase polymerase amplification.

In our assays we observed that the usage of 1% Cy5-dUTP produces very good results for the amplification efficiency as well as the labelling efficiency for a subsequent microarray analysis. Using higher amounts of the labelled nucleotides reduces the amount of products but on the other hand increases the number of incorporated fluorophores. This is not only indicated by the gel shift observed during gel electrophoresis but also by an increased fluorescence intensity after hybridization on a microarray.

The ratio of labelled and unlabelled nucleotides may be adjusted due to the experimental question under consideration. An increase of the amount of fluorophores also increases the background intensities, because unpurified RPA amplicons for the hybridization were used, producing unspecific absorptions of unused Cy5-dUTP to the glass surface.

In this study we decided to use a 1% Cy5-dUTP and 99% unlabelled dTTP as an appropriate ratio.

By using this labelling technique we were able to detect four different resistance genes (*bla*_CTX-M15_, *bla*_NDM_, *bla*_KPC_ and *bla*_VIM_) in *E. coli* and *P. aeruginosa*. The subsequent hybridization was very specific and none of the control probes nor the no-template-control showing false positives. We furthermore observed, that the fluorescence intensity in our assays depends strongly on the fragment length of the RPA products. The shortest fragment produced during the *CTX-M15* assay gives a 20 fold higher fluorescence signal compared to the largest fragment in the *KPC* assay. This indicates, that using short fragments with a high hybridization efficiency is more recommended than larger fragments with more fluorophores incorporated but a less hybridization efficiency. Similar results have been reported in the literature, evaluating the fragment conditions for microarrays^25^.

By the evaluation of a dilution series we were able to detect the *bla*_CTX-M15_ and *bla*_NDM_ resistance genes in genomic DNA corresponding to down to 228 cells and 380 cells respectively.

For diagnostics of antibiotic resistance genes it is sometimes helpful to detect not only the type of resistance genes but the correct variant, for example to develop epidemiological studies or to investigate the pathogen outbreak in detail^26^. Peter et al.^26^ has shown that it is possible to determine the gene variant with only one single mutation by using a very specific high signal probe. We also successfully detected not only *bla*_KPC_ but the *KPC-2* variant by using probes differing only in a single base pair. This result highlights the highly specificity of our novel RPA-labelling assay.

In this study we have shown that labelling of amplicons directly during the RPA reaction is a valuable alternative to pre-labelled primers and probes.

With this we have a novel tool for a flexible isothermal NAAT assay based on nucleotide labelling not only useful for laboratories but point-of-care diagnostics and lab-on-chip based systems. The current COVID-19 pandemic has shown, that “gold standard” NAATs like qPCR analysis are carried out basically in laboratories with trained staff. This results in processing times of more than 24 h for the whole process, including up to 3 h of nucleic acid amplification^4,27^.

As all PCR-based systems underlying the principal of thermocycling, with the need of fast and accurate temperature switching steps, PCR devices are often big with high demands of energy. Therefore, PCR depended systems are really sufficient and helpful in research but have disadvantages in diagnostics out of lab.

Using isothermal NAATs like RPA at constant temperatures of 37 °C reduces the PCR disadvantages to a minimum.

In this study, we were able to detect genomic DNA of 200–400 cells dependent on the organism and primer set used. As this represents successful results and high sufficiency for many of our questions, qPCR systems are able to operate with less amounts of DNA and to detect resistance gene of 1–20 cells per reaction^28,29^. We are hopefully able to increase our sensitivity in future studies.

In the case of specificity we were able to detect and distinguish amplicons only differing in one single nucleotide polymorphism (SNP). With this, we are as specific as modified qPCR system like DMAS-qPCR^30,31^ and more specific than SYBR green dependent qPCR reactions.

Here we describe a fast, sensitive and specific method with great potential as a point of care application by using pre-labelled nucleotides.

To date, RPA-based assays and devices capable for point of care applications (such as lateral flow assays) use pre-labelled primer^11,16^. For the combination of NAATs with the microarray technology and a possible detection of hundreds of target sequences with equally amounts of primer sets, it is in our opinion more useful to have a static labelling system. With this, we do not have the need of labelling every single primer pair.

Furthermore, labelling of nucleotides with other molecules is also possible. As this was a proof of concept study for the labelling with fluorophores we do need for the subsequent microarray analysis, there should be the possibility to use this technique for the labelling with various other molecules such as biotin-dNTPs, digoxigenin-dNTP or amine-modified nucleotides for subsequent custom labelling.

This should provide the ability to change the detection system as needed by simply changing one component in the reaction mixture rather than rebuying huge sets of oligonucleotides.

Even if the usage of other, probably simpler detection methods after RPA is possible, we decided to use microarray technology. With this method there is a capability to detect up to hundreds of different targets using only one and the same array format. For example, using LFA detection systems up to 3 analysis are detectable without much effort ^32^. In addition, differentiation of RPA products with SNPs is nearly impossible.

Summarizing, we have shown that labelling of RPA products and detection via microarray is a promising alternative to commonly used NAAT technologies. It combines the sensitivity of previous LFA or colorimetric based RPA assays and the specificity of probe dependent qPCR analysis. Beside this sensitivity and specificity it is flexible, easy to use and should be adaptable to various detection methods with different labelling molecules. Without the need of complex and expensive heating and cooling devices it is suitable for many point-of-care and lab-on-chip applications.

## Methods

### RPA reaction

In this study we used the TwistAMP® Liquid Basic Kit for DNA amplification (TwistDx™ Limited; TALQBAS01) according to the manufactures instructions with an amplification time of 40 min and a temperature of 38 °C. We performed a 25 µl reaction by downscaling the reaction reagents by 1:1 ratio. The DNA template consists of genomic DNA extracted, purified and provided by the german Robert Koch Institute as this was not included in this work (Table 1). Each reaction contains the two RPA-primers specific for the respective resistance gene (Table 2) and 1 µl of genomic DNA with varying concentrations depending on the experimental question. The relevant concentrations are indicated in the results section.

**Table 2:** List of immobilized probes.

Probes	Resistance gene	Sequence
CTX_M15_P200	*CTX-M15*	Spacer-GACTGGGTGTGGCATTGATTA
KPC_S_A	KPC*	Spacer-GATGACAAGAACAGCGAGG
KPC_S_C	KPC-2*	Spacer-GATGACAAGCACAGCGAGG
KPC_S_G	KPC*	Spacer-GATGACAAGGACAGCGAGG
KPC_S_T	KPC-3*	Spacer-GATGACAAGTACAGCGAGG
NDM_P_New	*NDM*	Spacer-GGACAAGATGGGCGGTAT
OXA_48_P1	*NC*	Spacer-CGCTCCGATACGTGTAACTTA
CTX_M14_P223	NC	Spacer-ACCAGTAAAGTTATGGCGGC
CTX_M14_P252	NC	Spacer-GCTTAAGCAGAGTGAAACGC
CTX_M14_P260	NC	Spacer-AGAGTGAAACGCAAAAGCAG
MCR_1_P437	NC	Spacer-ATTATCCGACTTGGGGCAAG

Illustration of the specific and unspecific (negative control; NC) probes used for the hybridization and detection of the RPA amplicons via microarray. The spacer consists of an aminohexyl-linker followed by an poly(T)-sequence. All probes were synthesized and provided by metabion international AG. (*reference: Peter et al.^26^).

For labelling the RPA amplicons and the following detection via microarray 0.5 µl (1 mM) 5-(3-Aminoallyl)-2′-deoxyuridine-5′-triphosphate labelled with Cy5 (Jena Bioscience, NU-803-XX-CY5-S) was added to the RPA reaction without changing the total reaction volume.

### RPA post-preparation and detection

For the detection of the RPA efficiency via gel electrophoresis, the products were purified with the Mag-Bind® Total Pure NGS Kit (Omega Bio-Tek; M1378-01) according to the manufactures instructions with a finishing eluation step in 15 µl ddH_2_O and 3 µl DNA Gel Loading Dye (6X) (Thermo Fisher Scientific; R0611) was added. An agarose gel with a 2% concentration in 1 × TAE-buffer (50 × TAE-buffer; PanReac AppliChem; A1691) was used for the separation of the fragments containing 2 µl of peqGreen (VWR; 732–3196) in a volume of 50 ml gel to visualize the band. A total volume of 18 µl of the purified and prepared samples were used for the electrophoresis which ran at 120 V for 1 h before the detection via BioDoc Analyzer (Biometra) including a Canon EOS 1100D was made.

The detection of the Cy5-dUTP labelled amplicons via microarray was performed without any purification of the RPA products.

### Microarray analysis

The hybridization of the labelled products and detection via microarray technology was carried out on a 3D-epoxy glass slide (PolyAn; 104 00 201) with immobilized target specific and unspecific probes (Table 2). For the immobilization the sciFLEXARRAYER SX (Scienion AG) was used with a 90 µm Piezo Dispense Capillary (PDC90) for the spotting of approximately 1 nL spotting solution for every single position at constant room conditions (75% rel. humidity and dew point cooling). The spotting solution consisted of 20 µM probes in a 1 × DNA spotting buffer (NEXTERION® Spot 2×; Schott; 1066029). Afterwards the slides were washed and blocked subsequently in 0.1% Triton X-100 (5 min), 6 mM HCl (4 min), 100 mM KCl (10 min), ddH_2_O (1 min), blocking solution (100 mM Tris-buffer, 50 mM ethanolamine, adjusting pH 9 with HCl; 15 min at 50 °C), ddH2O (1 min) and finally dried via nitrogen flow.

After the RPA reaction 5 µl hybridization buffer (20 × saline sodium citrate—SSC/ 0.6% Triton X-100 (v/v)) were added to the samples and the hybridization itself was performed in our self-developed hybridization chambers for 90 min at 52 °C followed by subsequent washing steps with 2 × SSC/0.2% sodium dodecyl sulphate—SDS (v/v) (10 min), 2 × SSC (10 min), 0.2 × SSC (10 min) and ddH_2_O for only one second. Finally, the glass slide was dried via nitrogen flow. All washing steps were done in beaker positioned on a magnetic stirrer at 300–500 rpm.

After the hybridization the microarrays were read out in a GenePix 4300 microarray scanner (Molecular Devices; Software GenePix Pro 7.2.28.003) with a scan power of 50–60% and a PMT gain of 500–600 to detect the Cy5 fluorescence at 635 nm excitation with a standard red emission filter.

## Data availability

The data that support the findings of this study are available from the corresponding author [C.W.] upon reasonable request.
